# Accelerometry-Based and Gyroscope-Based Detection of Sleep Apnea Events: Systematic Review of Technical and Clinical Evidence

**DOI:** 10.2196/104899

**Published:** 2026-10-22

**Authors:** Ruben Werle, Ruben Kuhlmann, Urs-Vito Albrecht

**Affiliations:** 1 Digital Medicine, Medical School OWL, Bielefeld University, Universitätsstraße 25, Bielefeld, North Rhine-Westphalia, 33615, Germany, 49 0521106 ext 86 7 14

**Keywords:** sleep apnea, obstructive sleep apnea, accelerometry, gyroscopy, inertial measurement units, wearable sensors, respiratory monitoring, apnea detection, polysomnography, systematic review

## Abstract

**Background:**

Sleep apnea is a common but frequently underdiagnosed disorder associated with increased cardiovascular and metabolic risk. Although polysomnography remains the diagnostic gold standard, its high cost, technical complexity, and limited availability restrict large-scale screening and continuous monitoring. Accelerometry-based and gyroscope-based approaches may represent a simplified alternative that can also be applied in home-based settings.

**Objective:**

This systematic review aimed to evaluate the technical and clinical evidence for accelerometry-based and gyroscope-based detection of apnea and hypopnea events and to assess their potential clinical applicability.

**Methods:**

A systematic literature search was conducted on April 12, 2025, in PubMed, Embase, Scopus, Web of Science, and IEEE Xplore following PRISMA (Preferred Reporting Items for Systematic Reviews and Meta-Analyses) 2020 guidelines. Studies investigating accelerometers, gyroscopes, inertial measurement units, seismocardiography, or ballistocardiography for apnea or hypopnea detection were included. Extracted data comprised study characteristics, sensor configurations, signal processing approaches, validation strategies, diagnostic performance, and clinical applicability. Methodological quality was assessed using the Quality Assessment of Diagnostic Accuracy Studies 2 (QUADAS-2) tool.

**Results:**

A total of 46 studies published between 2010 and 2025 were included. Most studies used accelerometers as the primary sensing modality, whereas gyroscopes were mainly applied in multimodal inertial measurement unit systems. Binary classification between apnea and nonapnea phases was the predominant analytical approach. Reported sensitivities ranged from 30% to 99%, depending on study settings, validation strategies, and evaluation levels. High diagnostic performance was mainly observed in simulation-based studies under controlled conditions. The included studies differed considerably in sensor placement, sampling frequencies, preprocessing pipelines, segmentation strategies, validation procedures, and reference standards. Only a minority of studies used polysomnography as the reference standard or included clinically representative populations. Furthermore, limited external validation, insufficient reproducibility, and a high risk of bias reduced comparability and clinical transferability.

**Conclusions:**

Current evidence supports the feasibility of accelerometer-based respiratory monitoring for sleep apnea screening, particularly in wearable and home-monitoring settings. However, most available studies were conducted under controlled experimental conditions and included only limited clinical validation. Standardized validation procedures and clinically representative cohorts are necessary to improve comparability and clinical transferability.

## Introduction

### Background

Sleep apnea is a common sleep-related breathing disorder characterized by recurrent interruptions of respiration during sleep. Obstructive sleep apnea (OSA) is associated with repetitive upper airway collapse, whereas central sleep apnea (CSA) is characterized by temporary cessation of respiratory effort [1]. Sleep-related breathing disorders are common in the general population, but many affected individuals remain undiagnosed [2,3]. The global burden of OSA is substantial: a literature-based analysis estimated that approximately 936 million adults aged 30 to 69 years have OSA, including approximately 425 million individuals with moderate-to-severe disease [4]. Despite this high prevalence, most cases are estimated to remain undiagnosed and untreated, underscoring the need for effective, accessible diagnostic strategies [4]. Untreated sleep apnea is associated with increased cardiovascular and metabolic morbidity and mortality, highlighting the importance of early diagnosis and monitoring [5,6]. Clinical assessment is commonly based on detecting apnea and hypopnea events and calculating the apnea-hypopnea index (AHI) [7]. Polysomnography is currently considered the diagnostic gold standard for sleep apnea assessment [8]. However, polysomnography requires specialized equipment, overnight monitoring, and trained personnel, resulting in high costs and limited accessibility. In addition, sleep laboratory environments may influence respiratory behavior and sleep quality, while long waiting times can delay diagnosis and treatment initiation [9,10]. To facilitate diagnosis outside the sleep laboratory, home sleep apnea testing (HSAT) has emerged as an established diagnostic option for appropriately selected patients with suspected OSA. The 2023 International Consensus Statement on OSA supports HSAT for patients with a high clinical probability of OSA and notes that treatment outcomes can be comparable to those following polysomnography-based diagnosis [11]. However, HSAT primarily serves to confirm, rather than reliably exclude, moderate-to-severe OSA, and negative, inconclusive, or technically inadequate studies may still require subsequent polysomnography [11]. These limitations, together with the substantial number of undiagnosed cases, have increased interest in simpler, wearable, and home-based approaches for respiratory monitoring and sleep apnea screening. Recent advances in wearable sensing technologies and AI have enabled increasingly automated approaches to sleep apnea detection. A recent systematic review and meta-analysis found promising diagnostic performance of wearable AI-based approaches, while also highlighting substantial heterogeneity in sensor modalities, signal types, algorithms, and study designs [10]. Inertial sensors, including accelerometers and gyroscopes, are of particular interest because they can capture respiratory-related thoracic and abdominal motion without requiring conventional physiological electrodes or pulse oximetry. Accelerometers detect linear motion, whereas gyroscopes detect rotational motion, enabling complementary characterization of respiratory mechanics. These technologies are already widely used in biomedical engineering applications, including movement analysis, cardiology, psychiatry, and digital health systems [12-16]

### Accelerometry and Gyroscopy for Respiratory Monitoring

Thoracic and abdominal breathing movements generate measurable acceleration and rotational signals that can be captured using wearable inertial sensors. Apnea episodes interrupt the periodic thoracic motion normally present during respiration, resulting in characteristic changes in accelerometer-derived signals [17]. The recorded signals can be processed using filtering, segmentation, feature extraction, and classification algorithms to identify apnea-related respiratory disturbances [18]. Recent studies have evaluated accelerometer-based and gyroscope-based systems for wearable and home-monitoring applications [19]. Their integration into smartphones, smartwatches, and wearable thoracic sensors enables unobtrusive respiratory monitoring over extended periods.

### Challenges and Research Gaps

Several methodological and clinical limitations currently restrict broader clinical implementation. Respiratory motion signals are highly susceptible to motion artifacts, changes in body position, and interindividual variability. Therefore, identifying the strengths of existing studies and evaluating their clinical transferability remain essential. Although previous systematic reviews have examined wearable technologies for sleep monitoring or broader applications of inertial sensors, a focused synthesis of accelerometry-based and gyroscope-based approaches for apnea and hypopnea event detection is still lacking. By integrating both the technical and clinical evidence, this review provides a comprehensive overview of current sensor technologies, signal processing methods, validation strategies, diagnostic performance, and the remaining challenges for clinical implementation.

### Objective

The objective of this systematic review was to critically evaluate the current technical and clinical evidence on accelerometry-based and gyroscope-based detection of apnea and hypopnea events. Specifically, we aimed to (1) summarize the sensor technologies, signal processing methods, and machine learning (ML) approaches used for event detection; (2) assess the available clinical validation and diagnostic performance of these approaches; and (3) identify current evidence gaps and future research needs to support the translation of wearable inertial sensing technologies into clinical practice.

## Methods

### Study Design

This systematic review was conducted and reported in accordance with the PRISMA (Preferred Reporting Items for Systematic Reviews and Meta-Analyses) 2020 guidelines [20] (Checklist 1). The review aimed to evaluate the current technical and clinical evidence regarding accelerometry-based and gyroscope-based detection of apnea and hypopnea events. The review protocol was not prospectively registered in a public repository, such as PROSPERO (International Prospective Register of Systematic Reviews), and no publicly accessible protocol was available. An internal protocol document was established before study selection and is available from the corresponding author upon reasonable request. However, the review methodology, eligibility criteria, search strategy, data extraction procedures, and quality assessment methods were defined before study selection and remained unchanged throughout the review process to minimize the risk of post hoc methodological modifications.

### Search Strategy

A systematic literature search was conducted on April 12, 2025, in the databases PubMed, Embase, Scopus, Web of Science, and IEEE Xplore. The systematic search was completed on April 12, 2025. Only studies identified through this search were eligible for inclusion in the systematic review. Publications published after the search date were cited selectively in the *Introduction* and *Discussion* sections to provide current context and were not included in the systematic evidence synthesis. The search strategy combined terms related to sleep apnea, hypopnea, respiratory monitoring, accelerometry, gyroscopy, inertial measurement units (IMUs), seismocardiography, and ballistocardiography. Different spellings and terminology variants related to apnea and hypopnea were included to maximize retrieval of relevant studies. The complete database-specific search strategies are provided in Multimedia Appendix 1.

### Eligibility Criteria

Studies were included if they (1) investigated human participants, (2) used accelerometry, gyroscopy, IMUs, or related inertial or motion-sensitive sensing approaches, (3) evaluated apnea and/or hypopnea events, (4) reported explicit differentiation between apnea/hypopnea and nonapnea events, and (5) were published as original journal or conference articles in English or German. Related approaches, including seismocardiography and ballistocardiography, were eligible if they used accelerometer-based or other inertial or motion-sensitive sensors to capture mechanically generated physiological signals relevant to apnea or hypopnea detection. These modalities were not considered interchangeable and were distinguished by the underlying sensing modality when sufficient information was available.

Studies were excluded if they involved animal experiments or in-vitro studies, did not use accelerometers, gyroscopes, IMUs, or related inertial or motion-sensitive sensing approaches, had no relation to apnea or hypopnea or an unclear thematic focus, did not explicitly record or evaluate apnea/hypopnea episodes, were not available as full text in English or German, or were reviews, comments, editorials, study protocols, letters to the editor, correspondence, or abstracts.

To provide a comprehensive overview of the current state of the field, studies with different experimental settings, including simulated apnea, voluntary breath-holding, respiratory monitoring, and clinically diagnosed sleep apnea, were included. Owing to their differing levels of clinical relevance, these study types were synthesized and interpreted separately.

### Study Selection

All identified records were imported into Rayyan (Qatar Computing Research Institute) for duplicate removal and study screening. Three reviewers independently screened titles and abstracts using predefined inclusion and exclusion criteria. Studies were included for full-text assessment if at least 2 reviewers supported inclusion. Full-text screening was subsequently performed by 2 reviewers, with uncertainties resolved through discussion and consensus within the review team. Reasons for exclusion were documented during full-text assessment and categorized according to the predefined eligibility criteria.

### Data Extraction

One reviewer extracted the data using a predefined, standardized extraction form; the extraction was verified by a second reviewer. Extracted variables included publication characteristics, study design, population characteristics, study setting, sensor technologies, sensor placement, sampling frequencies, preprocessing methods, segmentation approaches, feature extraction techniques, signal modalities, ML and deep learning (DL) models, validation strategies, reference standards, and diagnostic performance metrics. Additional variables included code availability, dataset availability, preregistration, funding sources, and conflicts of interest.

### Quality Assessment

Methodological quality and risk of bias were assessed using the Quality Assessment of Diagnostic Accuracy Studies 2 (QUADAS-2) tool [21]. Although QUADAS-2 was originally developed for diagnostic accuracy studies, it was applied pragmatically because the included literature comprised a heterogeneous mix of engineering, feasibility, and diagnostic studies, for which no single universally accepted quality assessment framework is available. The domains of patient selection, index test, reference standard, and flow and timing were evaluated for each included study. A formal or outcome-specific certainty-of-evidence assessment, such as GRADE (Grading of Recommendations Assessment, Development and Evaluation), was not performed because of the substantial heterogeneity in study design, sensor technologies, signal processing methods, reference standards, and reported outcome measures. The findings were therefore interpreted in the context of the methodological quality and risk-of-bias assessments, study designs, and validation approaches, without assigning formal certainty ratings.

### Data Synthesis

Because study populations, sensor configurations, signal processing pipelines, validation procedures, reference standards, and reported outcome measures varied considerably across studies, neither a quantitative meta-analysis nor meaningful subgroup analyses were considered appropriate. In many subgroups, the number of comparable studies was too small to support reliable quantitative comparisons. Therefore, the findings were synthesized narratively in accordance with the Synthesis Without Meta-analysis (SWiM) reporting guideline. Studies were grouped by study setting, sensor technology, and validation approach to identify common findings, differences, and evidence gaps. Particular attention was paid to distinguishing findings from simulation-based studies and clinically validated studies in patients with sleep apnea, given their differing levels of clinical relevance. Consequently, this review provides a structured synthesis and evidence mapping of the available literature rather than a quantitative assessment of diagnostic accuracy.

### Assessment of Reporting Bias

Potential reporting biases, including publication bias and selective outcome reporting, were considered qualitatively. Information on study preregistration, dataset availability, source code availability, funding sources, and conflicts of interest was extracted where reported. A formal statistical assessment of publication bias was not performed because of the substantial heterogeneity of the included studies and the absence of a quantitative meta-analysis. The potential influence of incomplete or selectively reported results was acknowledged as a limitation of the available evidence.

## Results

### Study Selection

The systematic literature search identified 2693 records. After duplicate removal, 1556 studies underwent title and abstract screening. Following full-text assessment (n=123), 46 studies published between 2010 and 2025 were included in the final analysis [22-67].

Of the 123 articles assessed in full text, 77 were excluded. The main reasons for exclusion were the absence of an accelerometer or gyroscope (n=18), absence of explicit apnea differentiation (n=17), publication as a conference abstract (n=15), review articles (n=1), studies not involving human participants (n=7), duplicate publications (n=6), and unavailable full text (n=13). These exclusion reasons are summarized in the PRISMA flow diagram (Figure 1).

**Figure 1.**
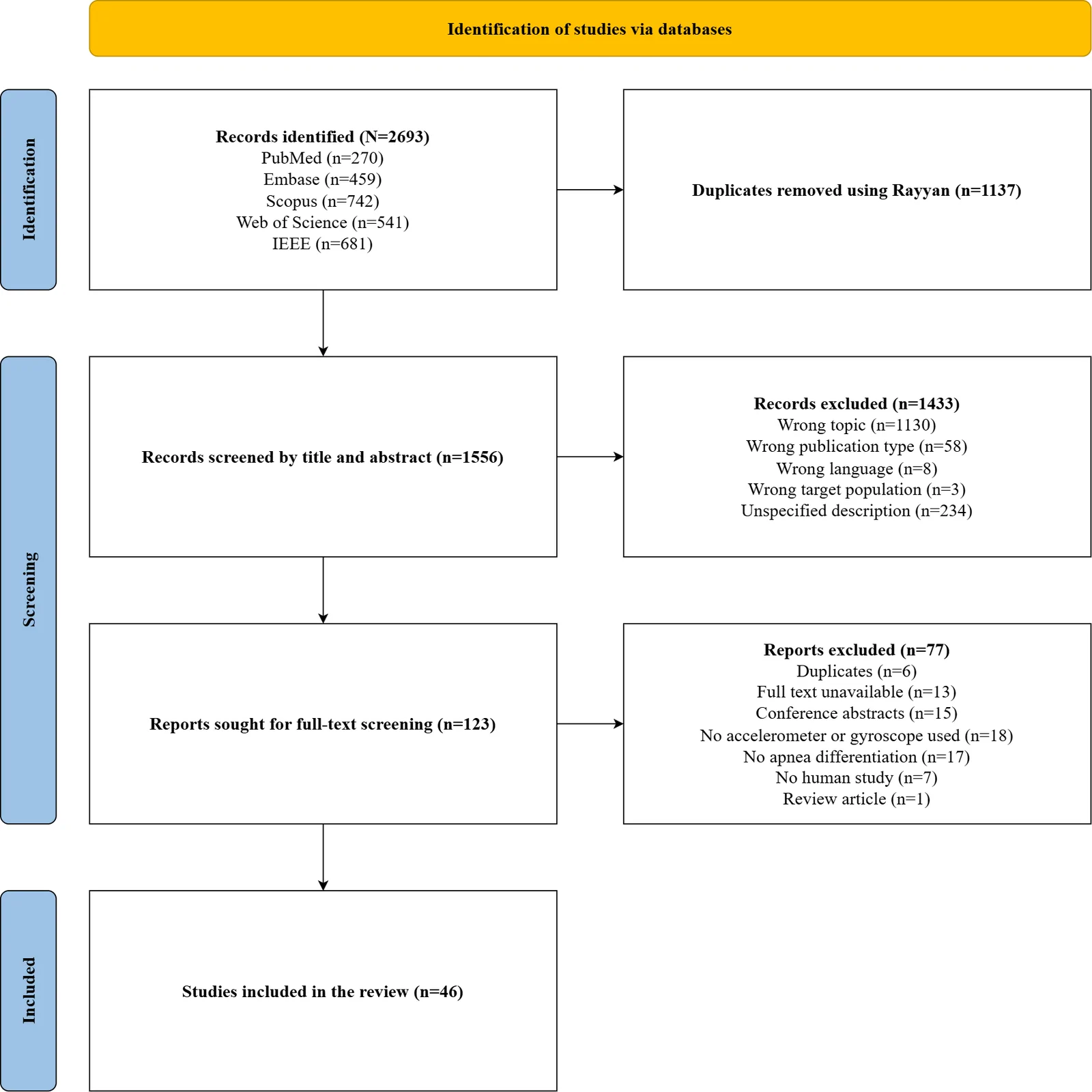
PRISMA (Preferred Reporting Items for Systematic Reviews and Meta-Analyses) 2020 flow diagram of the study selection process for the systematic review. A total of 46 studies were included after database searching, screening, and full-text assessment. Additional study selection and methodological details are provided in Multimedia Appendices 1-3. The PRISMA 2020 checklist is provided in Checklist 1.

### Study Characteristics

The included 46 studies differed markedly in study design, patient populations, sensor configurations, and validation procedures. Most studies were published as journal articles (n=32, 70%), whereas conference papers accounted for 14 (30%) studies. Most included studies were published after 2020. Geographically, studies were predominantly conducted in Europe (n=21, 46%) and North America (n=14, 30%). Simulation-based experimental settings dominated the literature (n=27, 59%), whereas studies conducted in sleep laboratories were less common (n=13, 28%). Real-world home-monitoring environments accounted for 3 (7%) studies, while clinical settings, including hospital environments, accounted for an additional 3 (7%) studies. Only a small number of studies included clinically representative populations of patients with sleep apnea. Women (n=19, 41%) and older adults (n=7, 15%) were underrepresented across many datasets. Polysomnography was used as the reference standard in only 12 (26%) studies. Many studies relied on simplified respiratory reference measures or did not clearly report a reference standard, limiting comparability and clinical interpretability. Detailed study characteristics of all included studies are summarized in Tables S1-S4 in Multimedia Appendix 2.

### Sensor Technologies and Measurement Setups

Accelerometers were the predominant sensing modality, used in most studies (29/46, 63%), whereas gyroscopes were primarily integrated into IMUs. Thoracic placement was the most common sensor location (18/46, 39%), although cervical, abdominal, smartwatch-based, and smartphone-based approaches were also investigated. Reported sampling frequencies ranged from 10 Hz in low-power wearable systems to 250 Hz in thoracic seismocardiography recordings and up to 10 kHz in vibration-focused laboratory measurements. Many systems combined inertial sensing with additional physiological signals such as electrocardiography, airflow monitoring, respiratory effort belts, or pulse oximetry. However, single-sensor wearable approaches remained common (19/46, 41%).

### Signal Processing and Modeling Approaches

Signal processing pipelines differed markedly between studies regarding filtering methods, segmentation approaches, feature extraction, and classification strategies. Preprocessing commonly involved bandpass (18/46, 39%) or low-pass filtering (13/46, 28%), while segmentation approaches ranged from sliding-window methods to event-triggered strategies. Morphological and time-domain features were most frequently extracted (19/46, 41%), whereas frequency-domain and nonlinear features were less common. Most studies focused on binary classification between apnea and nonapnea events (26/46, 57%). Rule-based and threshold-based methods were the most frequently used analytical approaches (11/46, 24%), followed by ML (5/46, 11%) and DL models (3/46, 7%). ML approaches primarily addressed apnea classification and AHI estimation, whereas DL architectures were increasingly used for automated feature extraction and multiclass classification tasks. Reported validation procedures ranged from simple train-test splitting to k-fold cross-validation, whereas external validation remained rare. More than half of the included studies did not clearly report a validation strategy. Detailed preprocessing pipelines, feature extraction approaches, analytical models, and validation strategies are summarized in Table S3 in Multimedia Appendix 2.

### Sensor Configuration and Placement

Across the included studies, thoracic and abdominal accelerometers were the most commonly investigated sensor configurations and consistently showed promising performance for detecting respiratory movements. While studies on ML approaches often report high diagnostic accuracy, meaningful comparisons between studies remain difficult due to methodological and validation differences. Evidence was strongest for studies validated against polysomnography, highlighting the need for further clinical validation of findings from simulation-based and voluntary breath-holding studies.

### Diagnostic Performance

#### Performance Across Evaluation Levels

Diagnostic performance varied across study settings and evaluation levels. Overall, event-level approaches tended to report higher and more consistent classification performance, whereas epoch-level and subject-level analyses showed greater variability. However, comparisons between settings should be interpreted with caution because the number of studies, reference standards, evaluation levels, and validation strategies varied substantially. Detailed diagnostic performance and reproducibility characteristics are provided in Table S4 in Multimedia Appendix 2.

#### Performance in Controlled Simulations

Controlled simulation studies comprised the largest group of included studies (27/46, 59%). These studies generally focused on detecting predefined respiratory events under controlled experimental conditions, with event-level analyses predominating (Table S6 in Multimedia Appendix 4). Among studies reporting diagnostic metrics, sensitivities ranged from 78.14% to 100% and specificities from 80% to 100%. Reported accuracies ranged from 85% to 97.50%. The highest performance was observed in event-level analyses, with sensitivities ranging from 78.14% to 100% and specificities of 100%. These findings support the technical feasibility of inertial-sensor–based respiratory event detection under controlled conditions.

#### Performance in Sleep Laboratory Settings

Among the 46 included studies, 13 (28%) studies were conducted in sleep laboratory settings, with several using polysomnography as the reference standard. Diagnostic performance was substantially more heterogeneous than in controlled simulations. Reported sensitivities ranged from 30.36% to 98%, while specificities ranged from 36% to 100%. Reported accuracies ranged from 69.11% to 96.9%. At the subject level, several studies focused on AHI-based severity classification and demonstrated performance that varied with the AHI threshold used. Epoch-level approaches likewise showed considerable variability, with lower performance reported in some validation settings (Table S6-S8 in Multimedia Appendix 4).

#### Performance in Home-Monitoring Environments

Among the 46 included studies, only 3 (7%) studies were conducted exclusively in home-monitoring environments, with 1 additional study combining simulation and home measurements. The limited number of studies precludes robust conclusions regarding diagnostic performance in real-world settings. Among the home-monitoring studies reporting diagnostic metrics, sensitivities ranged from 90% to 99.6%, with a reported specificity of 97.8% and an accuracy of 98.7% in one study (Table S6 in Multimedia Appendix 4). These apparently high values should be interpreted with caution, given the limited evidence base and differences in evaluation levels and reference standards.

### Risk of Bias and Reproducibility

QUADAS-2 assessment revealed substantial methodological limitations across 46 included studies. For patient selection, risk was rated low in 13 (28%) studies, unclear in 13 (28%), and high in 20 (43%), largely driven by case–control designs that were not avoided in 16 (35%) studies [24,28,32,39,40,46,49,51,56,58-60,63,65-67]. For the index test, 13 (28%) studies were assessed as having a low risk of bias, 8 (17%) as having an unclear risk, and 25 (54%) as having a high risk of bias. All high-risk assessments were attributable to a lack of blinding, as the reference standard was assessed with knowledge of the index test results [24,25,27,29,32,33,39,42,44,46,48-52,56,57,59,61-67]. For the reference standard, 12 (26%) studies were at low risk, 22 (48%) at unclear risk, and 12 (26%) at high risk, mainly because an inadequate gold standard was used (eg, simplified methods instead of polysomnography) [23,26-28,35,46-49,62,67]. Regarding flow and timing, 17 (37%) studies were at low risk, 20 (43%) at unclear risk, and 9 (20%) at high risk, most often due to incomplete inclusion of collected participant data [30,43,45,52,56,58,60,62,67]. Reproducibility was limited overall. Only a small number of studies provided publicly available datasets or source code, and preregistration was uncommon. External validation and clinically representative evaluation cohorts remained rare throughout the literature. Study-level QUADAS-2 assessments are provided in Multimedia Appendix 3.

## Discussion

### Principal Findings

This systematic review evaluated the current technical and clinical evidence regarding accelerometry-based and gyroscope-based detection of apnea and hypopnea events. The reviewed studies consistently showed that thoracic motion recorded by accelerometers can capture apnea-associated respiratory interruptions. While accelerometers were the predominant sensing modality [22-49,56], gyroscopes were mainly integrated into multimodal IMU systems [50-52,62]. Although this review also included gyroscope-based approaches, the available evidence for gyroscope-only systems remains limited. Most studies incorporated gyroscopes into multimodal IMU systems rather than evaluating their independent diagnostic value. Consequently, the current evidence does not allow firm conclusions regarding the specific contribution of gyroscopes to apnea detection. Most studies focused on binary classification between apnea and nonapnea events [24,26-30,35,38-40,44,46-51,53-61]. In contrast, hypopnea detection, differentiation between OSA and CSA, and estimation of AHI severity categories were investigated much less frequently. Consequently, the available evidence is strongest for binary apnea detection. Although several studies reported high classification performance, many investigations were performed under highly controlled experimental conditions. Existing studies varied considerably in study populations, sensor placement, preprocessing methods, feature extraction, validation strategies, and reference standards [22-67]. Although several studies reported high diagnostic performance, these findings were predominantly obtained under controlled simulation conditions rather than realistic clinical environments. Despite promising technical results, relatively few studies have evaluated these systems in clinically representative populations.

### Technical Feasibility vs Clinical Applicability

The distinction between technical feasibility and clinical applicability is particularly important when interpreting the diagnostic performance reported across the included studies. Most investigations used voluntary breath-holding or other experimentally controlled maneuvers to simulate apnea episodes [23,24,27-33,39,46,48,49,52,56-61,63-65,67]. Alternative approaches, including simulated OSA [50] and the Müller maneuver [62], were applied only sporadically. Notably, only one simulation study explicitly cited the American Academy of Sleep Medicine (AASM) criteria [23], underscoring the limited standardization of experimental apnea modeling across studies.

These controlled experimental designs provide important evidence that inertial sensors are technically capable of detecting respiratory interruptions. However, simulated apnea differs fundamentally from naturally occurring sleep apnea. During natural sleep, respiratory signals are affected by changes in body position, spontaneous movements, movement artifacts, irregular breathing patterns, and interindividual variability [54]. Simulation datasets therefore do not adequately reproduce the complex signal environment encountered during natural sleep. The consistently promising diagnostic performance observed in controlled experimental settings should consequently be interpreted primarily as evidence of technical feasibility rather than as an indicator of expected clinical performance. In contrast, studies conducted in sleep laboratories or home-monitoring environments demonstrated substantially greater variability in diagnostic performance [25,34,36,37,40,41,43,54,55,66]. In this context, Hayano et al [54] evaluated a wristwatch-integrated IMU combining triaxial accelerometry and gyroscopy against polysomnography in 122 adults. For the detection of moderate-to-severe sleep apnea (AHI≥15), the system achieved a sensitivity of 91% and a specificity of 78%, while severe sleep apnea (AHI≥30) was detected with a sensitivity of 90% and a specificity of 88%. These findings indicate that wearable IMU-based systems can provide clinically relevant information for sleep apnea screening. However, they should be interpreted within the broader evidence base, in which clinical and home-monitoring studies remain limited and heterogeneous. Another limitation is the predominance of small and clinically unrepresentative study populations. Many investigations included young (under 25) and healthy volunteers rather than clinically representative sleep apnea cohorts [24,49,52,54,57,59,62,64,66,67]. Women [22,23,27,28,36,41,42,44,46,50,54-56,58,60,64-67] and older adults [34,38,42,45,55] were frequently underrepresented. This limitation is consistent with recent evidence from OSA research, in which previous studies have predominantly included middle-aged to older men, potentially limiting the representation of OSA phenotypes in women [68]. Such demographic imbalances may limit the generalizability of reported diagnostic performance and highlight the need for validation in more diverse and clinically representative populations. For this reason, the reported performance values may not directly translate to routine clinical practice.

Limited interpretability of DL models may further complicate clinical implementation. DL approaches increasingly enable automated feature extraction and more complex classifications [26,38,51], but many architectures remain difficult to interpret clinically. In medical applications, limited explainability may reduce clinicians’ trust and complicate integration into clinical decision-making workflows. Explainable AI may improve transparency and should be prioritized in critical medical contexts [69].

Overall, these findings indicate that demonstrating technical feasibility under controlled conditions is an important first step, but robust validation under natural sleep and in real-world conditions is essential before such systems can be considered reliable tools for clinical sleep apnea screening.

### Methodological Considerations

The included studies differed markedly regarding sensor configurations, preprocessing pipelines, validation procedures, and reference standards. Accelerometer-based thoracic sensing represented the predominant measurement configuration, as thoracic displacement directly reflects respiratory motion [17]. This is supported by findings from Rendon et al [70], who evaluated different sensor placements and confirmed thoracic positioning as the most suitable approach. Additional sensor placements, particularly in the abdominal region, may further support differentiation between central and OSA [51]. Gyroscope-based approaches were comparatively uncommon and were mainly integrated into IMUs or multimodal sensor systems [50-52,62]. Other placements, such as the cervical or nasal regions, were used less frequently and primarily aimed at capturing respiratory vibrations or snoring-related signals. Sampling frequencies varied substantially, ranging from low-frequency respiratory motion tracking to high-frequency vibration analysis. While thoracic respiratory movements can be captured at relatively low sampling rates, higher sampling frequencies provide greater temporal resolution and may enable more precise signal processing, at the expense of increased computational and energy requirements. Recent work on wearable IMUs has demonstrated that the required sampling frequency depends strongly on the characteristics of the target movement, with lower sampling rates being sufficient for slower physiological or movement patterns, whereas higher rates may be required for faster dynamics [71]. The choice of sampling frequency therefore represents a trade-off between temporal resolution, signal quality, computational requirements, and energy consumption, particularly for long-term wearable monitoring. To mitigate motion artifacts, filtering and denoising techniques such as Butterworth filters were frequently applied. Despite existing recommendations, including those in the AASM Scoring Manual [7], no unified standard for sampling frequency has been established. Signal preprocessing typically included filtering (bandpass or low-pass) and segmentation using sliding-window, event-triggered, or physiologically triggered approaches. Feature extraction was primarily based on classical time-domain and morphological features derived from respiratory signals, whereas nonlinear and model-based features were used less frequently due to their higher complexity [35,42,46,47,49,67]. Some studies also included cardiac features [52,61,62,64,65], although these are not sufficiently specific on their own, as they are influenced by other physiological processes [72]. The analytical methods ranged from rule-based threshold systems to ML and DL approaches. Several rule-based approaches achieved high performance in controlled simulations [23,24,32,39,50]. ML performance was more variable and depended strongly on dataset composition, validation methodology, and feature extraction quality [32,35,37,45,46,55,63]. DL approaches increasingly enable automated feature extraction and more complex classifications [26,38,51], but their limited interpretability may restrict clinical applicability. As highlighted by Sands et al [73], differentiating between OSA and CSA remains one of the major challenges for automated detection systems. Interpretation of the current evidence is further complicated by variability in reference standards. Although polysomnography remains the clinical gold standard [8], only a minority of studies used polysomnography recordings for validation [22,34,36,37,41-45,54,55,66]. Several studies instead relied on simplified respiratory reference methods such as spirometry, airflow monitoring, or respiratory effort belts [23,26-28,35,46-49,62,67]. As a result, reported performance metrics cannot be easily compared across studies. Additional methodological details and study-specific analytical characteristics are summarized in Multimedia Appendix 2.

### Clinical Implications

The findings of this review suggest that accelerometry-based and gyroscope-based systems may support long-term respiratory monitoring in wearable and home-based settings. The widespread availability of microelectromechanical systems–based inertial sensors makes them attractive for wearable respiratory monitoring because they are small, inexpensive, and energy-efficient [74]. Their implementation in smartphones, smartwatches, or unobtrusive thoracic sensors may enable respiratory monitoring outside specialized sleep laboratories [25,36,38,53-55], which Park et al [75] described as a promising approach for sleep monitoring beyond the sleep laboratory environment. However, current evidence does not support replacement of polysomnography with inertial sensing technologies alone. Included studies remain limited by small sample sizes, nonrepresentative populations, heterogeneous methodologies, and insufficient clinical validation [22-67]. At present, accelerometry-based and gyroscope-based systems appear most suitable for supportive screening and longitudinal monitoring rather than standalone clinical diagnosis.

### Future Directions

Current studies differ substantially in sensor positioning, signal preprocessing, apnea definitions, validation procedures, and performance reporting, complicating direct comparisons between published systems. Overall, only a minority of the included studies were validated against polysomnography or evaluated in clinically representative sleep apnea populations. Most studies relied on simulation-based protocols, voluntary breath-holding, or small proof-of-concept cohorts, limiting the clinical generalizability of the reported diagnostic performance. Prospective validation studies using polysomnography as the reference standard in clinically representative OSA populations remain limited, and only a few studies have evaluated their methods on independent datasets. Future research should therefore focus on validating these approaches in larger and more heterogeneous patient cohorts, including real-world home-monitoring settings. In addition, binary classification approaches should be further evaluated for home-based screening, while more differentiated models that can distinguish apnea subtypes and separate apnea from hypopnea events are needed for reliable clinical diagnosis. Future studies should also improve the interpretability of DL models to facilitate their integration into clinical practice, as transparent decision-making is important for building clinician confidence in AI-assisted diagnostics. Standardized reporting of performance metrics, transparent descriptions of sensor configurations and signal processing methods, and external validation in independent cohorts would improve comparability across studies and strengthen the evidence base for the clinical translation of wearable inertial sensing technologies.

### Limitations

This review has several limitations. The included studies varied substantially in study populations, sensor technologies, signal processing methods, evaluation approaches, and validation procedures. These differences made a quantitative meta-analysis infeasible and necessitated a narrative synthesis of the findings. Variability in study quality, reference standards, and validation methodologies further limited direct comparability between studies. Furthermore, the review protocol was not prospectively registered, which may have reduced transparency regarding methodological decisions made during the review process. A formal assessment of evidence quality using GRADE was not performed because the evidence base was predominantly exploratory and technical and was not designed to inform clinical recommendations. Furthermore, methodological quality assessment using QUADAS-2 is partly interpretation-dependent and has limited applicability, as diagnostic accuracy studies comprised a minority of the included literature. The possibility of publication bias should also be considered, since studies with positive findings are generally more likely to be published. Given the absence of a formal statistical assessment, the extent to which selective publication or reporting influenced the available evidence remains uncertain. Reproducibility across the current literature also remains limited because only a minority of studies provided publicly available datasets, source code, or preregistered protocols. Supplementary methodological details and reproducibility characteristics are available in Multimedia Appendices 2 and 3.

### Conclusions

Current evidence indicates that accelerometer-based sensing can detect respiratory changes associated with sleep apnea. However, comparisons between studies remain difficult because of substantial methodological heterogeneity. Most published work still relies on simulation-based designs or small cohorts, limiting clinical generalizability. At present, these systems appear more suitable for screening and longitudinal monitoring than for standalone clinical diagnosis. Clinically representative, polysomnography-validated datasets remain limited, particularly for home-monitoring applications and longitudinal overnight recordings. Larger prospective studies with polysomnography as the reference standard in clinically representative populations are needed before these technologies can be recommended for diagnostic use.

## Supplementary material

10.2196/104899 Multimedia Appendix 1 Full search strategy.

10.2196/104899 Multimedia Appendix 2 Detailed study characteristics.

10.2196/104899 Multimedia Appendix 3 Quality Assessment of Diagnostic Accuracy Studies 2 (QUADAS-2) assessment.

10.2196/104899 Multimedia Appendix 4 Diagnostic performance tables.

10.2196/104899 Checklist 1 PRISMA 2020 checklist.

## Abbreviations

AASM: American Academy of Sleep Medicine
AHI: apnea-hypopnea index
CSA: central sleep apnea
DL: deep learning
GRADE: Grading of Recommendations Assessment, Development and Evaluation
HSAT: home sleep apnea testing
IMU: inertial measurement unit
ML: machine learning
OSA: obstructive sleep apnea
PRISMA: Preferred Reporting Items for Systematic Reviews and Meta-Analyses
PROSPERO: International Prospective Register of Systematic Reviews
QUADAS-2: Quality Assessment of Diagnostic Accuracy Studies 2
SWiM: Synthesis Without Meta-analysis
