# Supplementary material for: Accelerometry-Based and Gyroscope-Based Detection of Sleep Apnea Events: Systematic Review of Technical and Clinical Evidence
Source: JMIR Biomed Eng. 2026 Oct 22;11:e104899. doi: 10.2196/104899 (PMC13717323; doi:10.2196/104899)
Supplement: Supplementary file 1 [file biomedeng-v11-e104899-s001.docx]

# Multimedia Appendix 1. Full Search Strategy.

## PubMed (Search date: April 12, 2025)

((apnea[Title/Abstract]) OR (apnoea[Title/Abstract]) or (apnœa[Title/Abstract]) OR (apneic[Title/Abstract]) OR (apnoeic[Title/Abstract]) OR (apnœic[Title/Abstract]) OR (hypopn*a[Title/Abstract]) OR (hypopn*ic[Title/Abstract]) OR ((hold*[Title/Abstract]) AND (breath*[Title/Abstract])) OR (muller*[Title/Abstract]) OR (mueller*[Title/Abstract]) OR (valsalva[Title/Abstract]) OR (((breath*[Title/Abstract]) OR (inhal*[Title/Abstract]) OR (exhal*[Title/Abstract]) OR (inspir*[Title/Abstract]) OR (expir*[Title/Abstract]) OR (respir*[Title/Abstract])) AND ((manoeuvre*[Title/Abstract]) OR (maneuver*[Title/Abstract]) OR (maneuvre*[Title/Abstract])))) AND ((acceleromet*[Title/Abstract]) OR ((acceleration[Title/Abstract]) AND (sensor*[Title/Abstract])) OR (inertial[Title/Abstract]) OR (IMU[Title/Abstract]) OR (seismocardiogra*[Title/Abstract]) OR (ballistocardiogra*[Title/Abstract]) OR (kinetocardiogra*[Title/Abstract]) OR (kinocardiogra*[Title/Abstract]) OR (mechanocardiogra* [Title/Abstract]) OR (vibrocardiogra*[Title/Abstract]) OR (gyroscop*[Title/Abstract]) OR (gyrocardiogra*[Title/Abstract]) OR (gyrosensor*[Title/Abstract]) OR (gyrometer*[Title/Abstract]) OR (SCG[Title/Abstract]) OR (BCG[Title/Abstract]) OR (((seism*[Title/Abstract]) OR (ballist*[Title/Abstract]) OR (kinet*[Title/Abstract]) OR (kino*[Title/Abstract]) OR (mechan*[Title/Abstract]) OR (vibr*[Title/Abstract]) OR (gyro*[Title/Abstract])) AND (cardiogra*[Title/Abstract]))) NOT ((tuberculosis[Title/Abstract]) OR (bacillus[Title/Abstract]) OR (vaccine*[Title/Abstract]) OR (vaccination*[Title/Abstract]) OR (mice[Title/Abstract]) OR (rats[Title/Abstract]) OR (primates[Title/Abstract]))

## Embase (Search date: April 12, 2025)

(((apn*a):ti,ab,kw) OR ((apn*ic):ti,ab,kw) OR ((hypopn*a):ti,ab,kw) OR ((hypopn*ic):ti,ab,kw) OR ((hold* AND breath*):ti,ab,kw) OR ((muller*):ti,ab,kw) OR ((mueller*):ti,ab,kw) OR ((valsalva):ti,ab,kw) OR (((breath* OR inhal* OR exhal* OR inspir* OR expir* OR respir*) AND (manoeuvre* OR maneuver* OR maneuvre*)):ti,ab,kw)) AND (((acceleromet*):ti,ab,kw) OR ((acceleration AND sensor*):ti,ab,kw) OR ((inertial):ti,ab,kw) OR ((IMU):ti,ab,kw) OR ((seismocardiogra*):ti,ab,kw) OR ((ballistocardiogra*):ti,ab,kw) OR ((kinetocardiogra*):ti,ab,kw) OR ((kinocardiogra*):ti,ab,kw) OR ((mechanocardiogra*):ti,ab,kw) OR ((vibrocardiogra*):ti,ab,kw) OR ((gyroscop*):ti,ab,kw) OR ((gyrocardiogra*):ti,ab,kw) OR ((gyrosensor*):ti,ab,kw) OR ((gyrometer*):ti,ab,kw) OR ((SCG):ti,ab,kw) OR ((BCG):ti,ab,kw) OR (((seism* OR ballist* OR kinet* OR kino* OR mechan* OR vibr* OR gyro*) AND cardiogra*):ti,ab,kw) ) NOT (((tuberculosis):ti,ab,kw) OR ((bacillus):ti,ab,kw) OR ((vaccine*):ti,ab,kw) OR ((vaccination*):ti,ab,kw) OR ((mice):ti,ab,kw) OR ((rats):ti,ab,kw) OR ((primates):ti,ab,kw))

## Scopus (Search date: April 12, 2025)

( TITLE-ABS-KEY ( apn*a ) OR TITLE-ABS-KEY ( apn*ic ) OR TITLE-ABS-KEY ( hypopn*a ) OR TITLE-ABS-KEY ( hypopn*ic ) OR TITLE-ABS-KEY ( hold* AND breath* ) OR TITLE-ABS-KEY ( muller* ) OR TITLE-ABS-KEY ( mueller* ) OR TITLE-ABS-KEY ( valsalva ) OR TITLE-ABS-KEY ((breath* OR inhal* OR exhal* OR inspir* OR expir* OR respir*) AND ( manoeuvre* OR maneuver* OR maneuvre*) ) ) AND ( TITLE-ABS-KEY ( acceleromet* ) OR TITLE-ABS-KEY ( acceleration AND sensor*) OR TITLE-ABS-KEY ( inertial ) OR TITLE-ABS-KEY ( IMU ) OR TITLE-ABS-KEY ( seismocardiogra* ) OR TITLE-ABS-KEY ( ballistocardiogra* ) OR TITLE-ABS-KEY ( kinetocardiogra* ) OR TITLE-ABS-KEY ( kinocardiogra* ) OR TITLE-ABS-KEY ( mechanocardiogra* ) OR TITLE-ABS-KEY ( vibrocardiogra* ) OR TITLE-ABS-KEY ( gyroscop* ) OR TITLE-ABS-KEY ( gyrocardiogra* ) OR TITLE-ABS-KEY ( gyrosensor* ) OR TITLE-ABS-KEY ( gyrometer* ) OR TITLE-ABS-KEY (SCG) OR TITLE-ABS-KEY (BCG) OR TITLE-ABS-KEY (( seism* OR ballist* OR kinet* OR kino* OR mechan* OR vibr* OR gyro*) AND cardiogra*)) AND NOT (TITLE-ABS-KEY (tuberculosis) OR TITLE-ABS-KEY (bacillus) OR TITLE-ABS-KEY (vaccine*) OR TITLE-ABS-KEY (vaccination*) OR TITLE-ABS-KEY (mice) OR TITLE-ABS-KEY (rats) OR TITLE-ABS-KEY (primates))

## Web of Science (Search date: April 12, 2025)

(TS=(apn*a) OR TS=(apn*ic) OR TS=(hypopn*a) OR TS=(hypopn*ic) OR TS=(hold* AND breath*) OR TS=(muller*) OR TS=(mueller*) OR TS=(valsalva) OR TS=((breath* OR inhal* OR exhal* OR inspir* OR expir* OR respir*) AND (manoeuvre* OR maneuver* OR maneuvre*))) AND (TS=(acceleromet*) OR TS=(acceleration AND sensor*) OR TS=(inertial) OR TS=(IMU) OR TS=(seismocardiogra*) OR TS=(ballistocardiogra*) OR TS=(kinetocardiogra*) OR TS=(kinocardiogra*) OR TS=(mechanocardiogra*) OR TS=(vibrocardiogra*) OR TS=(gyroscop*) OR TS=(gyrocardiogra*) OR TS=(gyrosensor*) OR TS=(gyrometer*) OR TS=(SCG) OR TS=(BCG) OR TS=((seism* OR ballist* OR kinet* OR kino* OR mechan* OR vibr* OR gyro*) AND cardiogra*)) NOT (TS=(tuberculosis) OR TS=(bacillus) OR TS=(vaccine*) OR TS=(vaccination*) OR TS=(mice) OR TS=(rats) OR TS=(primates))

## IEEE Xplore (Search date: April 12, 2025)

("All Metadata":apn*a) OR ("All Metadata":apneic) OR ("All Metadata":apnoeic) OR ("All Metadata":apnœic) OR ("All Metadata":hypopnea) OR ("All Metadata":hypopnoea) OR ("All Metadata":hypopnœa) OR ("All Metadata":hypopneic) OR ("All Metadata":hypopnoeic) OR ("All Metadata":hypopnœic) OR ((("All Metadata": hold) OR ("All Metadata": holds) OR ("All Metadata": holding)) AND (("All Metadata": breath) OR ("All Metadata": breathing))) OR ("All Metadata":muller) OR ("All Metadata":mueller) OR ("All Metadata":muller’s) OR ("All Metadata":mueller’s) OR ("All Metadata":valsalva) OR ((("All Metadata": breath) OR ("All Metadata": breathing) OR ("All Metadata": inhalation) OR ("All Metadata": exhalation) OR ("All Metadata": inspiration) OR ("All Metadata": inspiratory) OR ("All Metadata": expiration) OR ("All Metadata": expiratory) OR ("All Metadata": respiration) OR ("All Metadata": respiratory)) AND (("All Metadata": manoeuvre) OR ("All Metadata": manoeuvres) OR ("All Metadata": maneuver) OR ("All Metadata": maneuvers) OR ("All Metadata": maneuvre) OR ("All Metadata": maneuvres)))) AND (("All Metadata":acceleromet*) OR (("All Metadata": acceleration) AND (("All Metadata": sensor) OR ("All Metadata": sensors))) OR ("All Metadata": inertial) OR ("All Metadata": IMU) OR ("All Metadata": seismocardiogra*) OR ("All Metadata": ballistocardiogra*) OR ("All Metadata": kinetocardiogra*) OR ("All Metadata": kinocardiogra*) OR ("All Metadata": mechanocardiogra*) OR ("All Metadata": vibrocardiogra*) OR ("All Metadata": gyroscop*) OR ("All Metadata": gyrocardiogram) OR ("All Metadata": gyrocardiograms) OR ("All Metadata": gyrocardiography) OR ("All Metadata": gyrocardiographic) OR ("All Metadata": gyrosensor) OR ("All Metadata": gyrosensors) OR ("All Metadata": gyrometer) OR ("All Metadata": gyrometers) OR ("All Metadata": SCG) OR ("All Metadata": BCG) OR ((("All Metadata": seismo) OR ("All Metadata": seismic) OR ("All Metadata": ballisto) OR ("All Metadata": ballistic) OR ("All Metadata": kineto) OR ("All Metadata": kinetic) OR ("All Metadata": kino) OR ("All Metadata": mechano) OR ("All Metadata": mechanic) OR ("All Metadata": vibro) OR ("All Metadata": vibrational) OR ("All Metadata": gyro*)) AND (("All Metadata": cardiography) OR ("All Metadata": cardiogram) OR ("All Metadata": cardiographic))) NOT (("All Metadata": tuberculosis) OR ("All Metadata": bacillus) OR ("All Metadata": vaccine) OR ("All Metadata": vaccines) OR ("All Metadata": vaccination) OR ("All Metadata": vaccinations) OR ("All Metadata": mice) OR ("All Metadata": rats) OR ("All Metadata": primates))
