# Supplementary material for: Accelerometry-Based and Gyroscope-Based Detection of Sleep Apnea Events: Systematic Review of Technical and Clinical Evidence
Source: JMIR Biomed Eng. 2026 Oct 22;11:e104899. doi: 10.2196/104899 (PMC13717323; doi:10.2196/104899)
Supplement: Supplementary file 2 [file biomedeng-v11-e104899-s002.docx]

# Multimedia Appendix 2. Detailed Study Characteristics.

This appendix provides detailed characteristics of all included studies, including publication year, study setting, participant characteristics, sensor technologies, sensor placement, sampling frequencies, preprocessing approaches, signal modalities, analytical methods, validation strategies, reference standards, and reported diagnostic performance metrics. The extracted variables additionally include information regarding machine learning and deep learning approaches, feature extraction methods, reproducibility measures, and risk-of-bias–relevant methodological aspects. Detailed study characteristics are summarized in Tables S1-S4.

Table S1. General Characteristics of Included Studies

| **Study** | **Author** | **Year** | **Country** | **Study Setting** | **Population** | **Sample Size** | **Reference Standard** |
| --- | --- | --- | --- | --- | --- | --- | --- |
| [20] | Hafezi et al. | 2019 | Canada | Sleep laboratory | Sleep apnea | 20 | PSG |
| [21] | Boiko et al. | 2023 | Germany | Simulation | Healthy | 3 | Respiratory effort belt |
| [22] | Pierleoni et al. | 2014 | Italy | Simulation | Healthy | 20 | None |
| [23] | Guul et al. | 2016 | Denmark | Home | Mixed | 3 | None |
| [24] | Micheaux et al. | 2023 | France | Sleep laboratory | Mixed | 28 | Nasal cannula |
| [25] | Dehkordi et al. | 2012 | Canada | Simulation | Healthy | 19 | Nasal/oral flow |
| [26] | Pandia et al. | 2013 | USA | Simulation | Healthy | 20 | Respiratory effort belt |
| [27] | González et al. | 2015 | Spain | Simulation | Healthy | 4 | None |
| [28] | Phan et al. | 2017 | Vietnam | Simulation | N/A | 5 | None |
| [29] | Akbugday et al. | 2019 | Turkey | Simulation | Healthy | 1 | None |
| [30] | Lee et al. | 2015 | South Korea | Simulation | N/A | 20 datasets | None |
| [31] | Hung et al. | 2018 | Vietnam | Simulation | N/A | 30 | None |
| [32] | Hafezi et al. | 2020 | Canada | Sleep laboratory | Sleep apnea | 80 | PSG |
| [33] | Yüzer et al. | 2020 | Turkey | Sleep laboratory | Sleep apnea | 5 | Spirometry |
| [34] | Ferrer-Lluis et al. | 2020 | Spain | Home | Mixed | 13 | AHI ApneaLink |
| [35] | Bricout et al. | 2021 | France | Sleep laboratory | Sleep apnea | 28 | PSG |
| [36] | Liu et al. | 2024 | USA | Sleep laboratory | Sleep apnea | 20 | None |
| [37] | Kim et al. | 2014 | South Korea | Simulation | N/A | 20 datasets | None |
| [38] | Manoni et al. | 2020 | Italy | Hospital | Sleep apnea | 1 | None |
| [39] | Hsu et al. | 2020 | Taiwan | Sleep laboratory | Sleep apnea | 119 | PSG |
| [40] | Kim et al. | 2024 | South Korea | Sleep laboratory | Mixed | 50 | PSG |
| [41] | Saha et al. | 2020 | Canada | Sleep laboratory | Sleep apnea | 85 | PSG |
| [42] | Morillo et al. | 2010 | Spain | Sleep laboratory | OSA | 15 | PSG |
| [43] | Pandia et al. | 2012 | USA | Simulation | Healthy | 20 | None |
| [44] | Sweeney et al. | 2013 | Ireland | Hospital | Sleep apnea | 5 | PSG |
| [45] | Azad et al. | 2019 | USA | Simulation | Healthy | 5 | Spirometry |
| [46] | Yüzer et al. | 2020 | Turkey | Home | Sleep apnea | 10 | Spirometry |
| [47] | Das et al. | 2023 | Canada | Simulation | N/A | 1 | Spirometry |
| [48] | Ahdy et al. | 2024 | USA | Simulation | Healthy | 15 | Spirometry |
| [49] | Thuong et al. | 2019 | Vietnam | Simulation | Healthy | 5 | None |
| [50] | McClure et al. | 2020 | USA | Simulation | Healthy | 100 | None |
| [51] | Morra et al. | 2019 | Belgium | Simulation | Healthy | 49 | None |
| [52] | Morra et al. | 2020 | Belgium | Simulation | Healthy | 49 | Spirometry |
| [53] | Senigagliesi et al. | 2022 | Italy | Simulation | Healthy | 2 | None |
| [54] | Gambi et al. | 2024 | Italy | Simulation | Healthy | 2 | None |
| [55] | Hayashi et al. | 2024 | Malaysia | Simulation | Healthy | 20 | None |
| [56] | Hassan et al. | 2021 | USA | Simulation | Healthy | 8 | None |
| [57] | Cámara et al. | 2017 | Spain | Simulation/Home | Mixed | 4 | None |
| [58] | Hayano et al. | 2024 | Japan | Sleep laboratory | Sleep apnea | 122 | PSG |
| [59] | Hayano et al. | 2025 | Japan | Sleep laboratory | Sleep apnea | 68 | PSG |
| [60] | García Cena et al. | 2023 | Spain | Simulation | Mixed | 35 | None |
| [61] | Nitta et al. | 2024 | Japan | Simulation | Tumor patients | 26 | None |
| [62] | Ryser et al. | 2022 | Switzerland | Sleep laboratory | Healthy | 13 | PSG |
| [63] | Skoric et al. | 2022 | Canada | Simulation | Healthy | 40 | Spirometry |
| [64] | Skoric et al. | 2019 | Canada | Simulation | Healthy | 24 | None |
| [65] | Morra et al. | 2020 | Belgium | Simulation | Healthy | 28 | None |

Table S2. Sensor Technologies and Measurement Configurations

| **Study** | **Sensor Type** | **Additional Sensors** | **Sensor Placement** | **Sampling Frequency** | **Wearable System** |
| --- | --- | --- | --- | --- | --- |
| [20] | Accelerometer | None | Neck | Not reported | Yes |
| [21] | Accelerometer | None | Bed-based / contactless | 62 Hz | No |
| [22] | Accelerometer | Pressure sensor | Thorax / chest | 50 Hz | Yes |
| [23] | Accelerometer | Audio | Thorax / chest | 10 Hz | Yes |
| [24] | Accelerometer | ECG | Thorax + abdomen | 256 Hz | Yes |
| [25] | Accelerometer | Respiratory effort sensor | Neck + thorax + abdomen | 500 Hz | Yes |
| [26] | Accelerometer | ECG | Thorax / chest | Not reported | Yes |
| [27] | Accelerometer | None | Thorax / chest | 16 Hz | Yes |
| [28] | Accelerometer | Respiratory inductance plethysmography (RIP) | Thorax / chest | 1000 Hz | Yes |
| [29] | Accelerometer | None | Abdomen | Not reported | Yes |
| [30] | Accelerometer | ECG | Thorax / chest | 4 Hz | Yes |
| [31] | Accelerometer | ECG | Thorax / chest | 1000 Hz | Yes |
| [32] | Accelerometer | None | Neck | 60 Hz | Yes |
| [33] | Accelerometer | None | Abdomen | 20 Hz | Yes |
| [34] | Accelerometer | None | Thorax / chest | 200 Hz | Yes |
| [35] | Accelerometer | None | Thorax + abdomen | 256 Hz | Yes |
| [36] | Accelerometer | None | Wrist | 40-60 Hz | Yes |
| [37] | Accelerometer | ECG | Thorax / chest | 1 Hz | Yes |
| [38] | Accelerometer | PPG | Nose | 16 Hz | Yes |
| [39] | Accelerometer | ECG | Thorax / chest | 31.25 Hz | Yes |
| [40] | Accelerometer | EEG | Thorax / chest | 50 Hz | Yes |
| [41] | Accelerometer | Pulse oximetry, Respiratory sound data | Neck | 60 Hz | Yes |
| [42] | Accelerometer | None | Neck | 8192 Hz | Yes |
| [43] | Accelerometer | Respiratory effort belt, ECG | Thorax / chest | 10000 Hz | Yes |
| [44] | Accelerometer | Respiratory effort belt, ECG | Thorax / chest | 25 Hz | Yes |
| [45] | Accelerometer | ECG | Thorax / chest | 10000 Hz | No |
| [46] | Accelerometer | None | Abdomen | 20 Hz | Yes |
| [47] | Accelerometer | Pressure sensor | Back / lumbar region | 100 Hz | Yes |
| [48] | Accelerometer | ECG | Thorax / chest | 1000 Hz | No |
| [49] | Gyroscope + accelerometer | None | Neck | Not reported | Yes |
| [50] | Gyroscope + accelerometer | None | Thorax + abdomen | 125 Hz | Yes |
| [51] | Gyroscope + accelerometer | ECG | Back / lumbar region + thorax / chest | 50 Hz | No |
| [52] | Gyroscope + accelerometer | ECG, Pulse oximetry | Back / lumbar region | 2-20 Hz | No |
| [53] | BCG | None | Bed-based / contactless | 1000 Hz | No |
| [54] | BCG | None | Bed-based / contactless | 1000 Hz | No |
| [55] | BCG | EEG | Not reported | 1000 Hz | No |
| [56] | SCG | ECG, Spirometry | Thorax / chest | 10000 Hz | No |
| [57] | IMU | Audio | Thorax / chest | max. sampling rate | Yes |
| [58] | IMU | None | Wrist | 32 Hz | Yes |
| [59] | IMU | None | Wrist | 60 Hz | Yes |
| [60] | IMU | None | Thorax + abdomen | 100 Hz | Yes |
| [61] | Gyroscope | Respiratory Gating (RGSC) | Abdomen | 100 Hz | No |
| [62] | IMU | None | Thorax / chest | 50 Hz | Yes |
| [63] | IMU | ECG, Pneumotachograph | Abdomen | 600 Hz | Yes |
| [64] | IMU | ECG | Thorax / chest | 280 Hz | Yes |
| [65] | BCG/SCG | ECG, Blood pressure monitor, Pulse oximetry, Respiratory effort belt | Back / lumbar region + thorax / chest | 50 Hz | Yes |

Table S3. Signal Processing and Classification Approaches

| **Study** | **Preprocessing** | **Segmentation** | **Feature Extraction** | **Classification Approach** | **Validation Strategy** |
| --- | --- | --- | --- | --- | --- |
| [20] | 3rd-order Butterworth band-pass filter (0.1–25 Hz) | Sliding window of 10 s with 9 s overlap | Respiratory cycle features: mean signal (inspiration to expiration), mean breathing duration, distance between the minimum of the current window and the maximum of the previous window, ratio of inspiration time to breathing duration, area under the inspiratory curve, rise rate, total area under the respiratory signal | Deep Learning: convolutional neural network, recurrent neural network, and their combination | Hold-out / Train-Test |
| [21] | 4th-order Butterworth low-pass filter (0.4 Hz) | Not reported | Signal peaks | Threshold-based algorithm | None reported |
| [22] | 4th-order Butterworth low-pass filter (0.45 Hz); Fast Fourier Transform (FFT) | Window length of 10.08 s with 1.008 s overlap | Estimation of respiratory rate | Rule-based algorithm | External validation |
| [23] | 6th-order Butterworth band-pass filter (0.1–0.8 Hz); axis fusion (X, Y, Z); upsampling | Frames of 0.25 s duration | Heart rate, respiratory rate, sleep position, apnea events | Rule-based approach (MATLAB) | External validation |
| [24] | Resampling; Z-score normalization; shuffling; removal of overrepresented classes; splitting into training, validation, and test data | 12 s windows with 11 s overlap | Abdominal and thoracic respiratory effort | Deep learning: MLP, CNN, LSTM and GRU | None reported |
| [25] | Band-pass filter (0.1–10 Hz); downsampling to 20 Hz; Savitzky-Golay filter (window size: 100 ms, 2nd-order polynomial) | Trigger-based (oral-nasal flow): segmentation into tidal and deep segments | Respiratory flow and breathing effort | Ensemble Learning: Non-causal FIR model and neural network model | External validation |
| [26] | Low-pass filter (100 Hz), high-pass filter (0.5 Hz), notch filter (60 Hz) | Continuous windows (based on the mean breathing interval) | Power spectrum | Visual and statistical comparison | None reported |
| [27] | Band-pass filter (low and high frequency ranges) | Continuous window of length 16 with a shift of one sample | Body acceleration | Threshold based algorithm | None reported |
| [28] | Adaptive band-pass filter | 60 s windows with 10 s overlap | Waveform-based features and respiratory rate | Algorithmic approach | External validation |
| [29] | On-board low-pass filter (5 Hz) with prior calibration | Continuous segmentation | Breathing patterns, breath count, respiratory rate, apnea episodes | System-on-Chip (SoC) analysis: apnea detection based on reduced abdominal movement | None reported |
| [30] | Low-pass filter (1 Hz); high-pass filter (0.1 Hz) | Hamming window of size 64 with an overlap of 63 samples | LF/HF-based and acceleration features | Analysis with Weka; AdaBoost algorithm | 10-fold cross-validation |
| [31] | Not reported | Not reported | Histogram ratios, spectral ratios, wavelet coefficients, linear prediction coefficients | Artificial neural network: Multilayer Perceptron type with two hidden layers | 10-fold cross-validation |
| [32] | 3rd-order Butterworth band-pass filter (0.1–25 Hz) | Sliding window of 10 s with 9 s overlap | Respiratory cycle features | Deep Learning: combination of convolutional neural network, Long Short-Term Memory (LSTM) layer, and fully connected layer | 5-fold cross-validation: two loops |
| [33] | Moving average filter (window size: 10) as a low-pass filter | Not reported | Model parameters (i, j, k as inputs; b1, bk as bias; w1, w2 as weights) | Artificial neural network: V7.12.0.635 (R2011a) Matlab software version which has Neural Network Toolbox | External validation |
| [34] | Artifact removal; low-pass filtering (0.8 Hz) using an 8th-order Butterworth filter | Overlapping 45 s segments, shifted sample by sample | Signal-based coefficients for evaluating angular variability (relative to previous and subsequent performance) | Threshold-based approach according to AASM hypopnea criteria (≥70% reduction) | None reported |
| [35] | Band-pass filter (0.05–0.8 Hz); signal reconstruction | Not reported | Statistical and spectral features per epoch: median, standard deviation, coefficient of variation, mean/median absolute deviation, kurtosis, interquartile range, dispersion metric, VLF, LF, and HF power, LF/HF ratio, spectral kurtosis, respiratory rate, aperiodic rate, energy rate, variance of the dispersion metric | Machine learning: RUSBoosted Trees | Leave-One-Out Cross Validation |
| [36] | Total variation filter; linear weighting mechanism | Not reported | CNN-based feature extractor | Custom Deep Learning Model (CNN + Self-Attention + Cross-Axis Correlation) | None reported |
| [37] | Not reported | Not reported | LF/HF-based features: maximum, minimum, and mean LF/HF ratio; mean acceleration values in x-, y-, and z-axis | AdaBoost (boosting algorithm) | 10-fold cross-validation |
| [38] | Windowing, artifact removal, 4th-order Butterworth filter (double-filter technique analogous to PPG); bandwidth: 0.2–3.5 Hz | Event-based: only during apnea detected by PPG | Power spectral density (PSD) | Rule- and threshold-based approach (flag cross-checking) | None reported |
| [39] | Butterworth band-pass filter (0.15–0.4 Hz); downsampling to 1 Hz; peak detection | Sliding window of 5 s | Chest Effort Index, CVHR | Statistical analysis (DeLong test) | None reported |
| [40] | 1st-order band-pass filter | Segmentation analogous to PSG (10 s windows) | Statistical features (mean, standard deviation, skewness, kurtosis) for X, Y, Z and their sum; correlation features between axes; crest factors of the respiratory waveform | Stacking ensemble with SVM, logistic regression (LR), and multilayer perceptron (MLP) | 10-fold cross-validation |
| [41] | Low-pass filter (0.5 Hz) | 10 s windows with 80% overlap | Mean respiratory energy, proportion of respiratory duration, reduction of respiration-related movements (X, Z) at event onset, decrease in SpO₂ | Weighted feature aggregation and event-based classification using thresholds | 3-fold cross-validation |
| [42] | 30th-order low-pass FIR filter | Not reported | Airflow and acceleration data | Rule-based algorithm | None reported |
| [43] | Low-pass filter (50 Hz); Savitzky-Golay polynomial approximation with subtraction; denoising using Daubechies wavelet (4th order, 14 decomposition levels, soft thresholding) | Beat-based segmentation (heartbeats) | Heart sound features: S1–S1 interval, intensity ratio, S1–S2 interval | Student’s t-test with Holm–Bonferroni correction | External validation |
| [44] | 2nd-order Butterworth band-pass filter, low-pass filter (0.8 Hz); normalization; discrete wavelet transform | Individual apnea events: 20 s windows | Six features per data window and signal modality | Decision tree classifier | 10-fold cross-validation |
| [45] | 4th-order Chebyshev Type II band-pass filter (0.5–50 Hz) with 15 dB stopband attenuation; correction of respiratory baseline wander (<0.5 Hz); 5th-order moving average filter | R-peak-based segmentation: each SCG segment starts 0.1 s before the corresponding ECG R-peak and ends 0.1 s before the next R-peak | SCG waveform | Unsupervised machine learning: k-medoids clustering with Dynamic Time Warping (DTW) | None reported |
| [46] | Offset-based sampling filtering | Not reported | Diaphragm movement | Threshold-based approach | None reported |
| [47] | Type II Chebyshev low-pass filter (LPF) with cutoff frequency of 150 Hz; peak detection | Not reported | Body movement | Peak detection analysis: MATLAB (MathWorks, R2021a) | None reported |
| [48] | Chebyshev Type II band-pass filter (0.05–200 Hz); downsampling of segmented beats to 500 Hz; normalization by mean subtraction and scaling to peak-to-peak amplitude | Beat-based segmentation: starts 100 ms before the corresponding R-peak and ends 100 ms before the subsequent R-peak | SCG waveform (intra- and inter-state variability, frequency analysis) | Machine learning: K-medoid algorithm | None reported |
| [49] | Heart rate (HR): Savitzky-Golay low-pass filter and peak detection | Event-based: based on the dominant signal (10 s windows) | Heart rate and bicoherence | Bispectral analysis in combination with an algorithmic approach | None reported |
| [50] | Not reported | Event-based (breathing, 7 s windows) | 12 raw features | Convolutional Neural Network (CNN, deep learning) | None reported |
| [51] | R-peak detection; manual identification of breathing maneuvers; baseline definition | Window width of 10 s | Kinetic energy (systole and diastole) | Statistical analysis (SPSS) | None reported |
| [52] | R-peak detection; manual identification of breathing maneuvers | Event-based with 10 s windows | Kinetic energy (total) | Statistical analysis | None reported |
| [53] | 6th-order Butterworth low-pass filter (0.7 Hz) | Not reported | Mean R–R interval | Automatic algorithm: apnea detection for event duration > 20 s | None reported |
| [54] | 6th-order Butterworth low-pass filter (0.7 Hz) | Not reported | Mean R–R interval | Automatic algorithm: apnea detection for event duration > 20 s | None reported |
| [55] | Normalization to range [-1, 1]; amplitude scaling to 1 | Not reported | Amplitude and cycle changes | Neural Network: 1D-CNN | None reported |
| [56] | Downsampling to 1000 Hz; band-pass filter (0.05–200 Hz) | Event-based: segmentation by cardiac cycles | Heart rate (HR), heart rate variability (HRV), SCG waves | Unsupervised machine learning (k-medoids) and Support Vector Machine (SVM) | None reported |
| [57] | Linear resampling to 200 Hz | Not reported | Respiratory cycles and apnea events | Not reported | None reported |
| [58] | Band-pass filter (0.13–0.7 Hz); combination of the three axes | Sliding windows with variable length of 3–30 s | Respiratory Event Index, sleep apnea episodes | Statistical analysis and rule-based approach | Hold-out / Train-Test |
| [59] | Band-pass filter (4–11 Hz) | Sliding segments of 32 s | Frequency Stability Index (FSI) | Feature-based Machine Learning classifier (15 handcrafted IMU features; binary classification) | Hold-out / Train-Test |
| [60] | Kalman filter in combination with low-pass filter, Savitzky-Golay smoothing, Z-score normalization, peak detection | Not reported | RR and respiratory dynamics: mean, standard deviation, maximum/minimum inspiration time, maximum expiration time, peak metrics (max., min., means), entropy | Automated calculation in MATLAB (Human–Machine Interface) | External validation |
| [61] | Min-max normalization (minimum value = 0) | Segment definition based on respiratory signal | Respiratory waveform, success rate of expiratory breath-holding | Mann–Whitney U test (SPSS 8.0) | Hold-out / Train-Test |
| [62] | Zero-phase Butterworth high-pass filter (IIR), 29th order (0.05 Hz), followed by a zero-phase Butterworth low-pass filter (IIR), 10th order (1.0 Hz) | Epochs of 12 s duration | Respiration-related and statistical features: respiratory rate (RR), peak variance, standard deviation, variance, peak-to-peak difference, peak-to-RMS ratio, peak-to-average signal/power, kurtosis, cross-correlations (x–y, x–z, y–z), median and mean frequency, SINAD, bandpower | Machine learning approach: K-NN and RUSBoost | 10-fold cross-validation |
| [63] | Amplitude and frequency modulation; correction of baseline wander | Not reported | X-axis baseline, heart rate, AO amplitude, IC amplitude | Statistical analysis and rule-based approach | External validation |
| [64] | ECG-based R-peak detection for heartbeat identification | Shiftable windows with 15 samples (≈ 60 ms) | Root-mean-square (RMS) of the averaged waveform | Envelope function in MATLAB | External validation |
| [65] | High-pass filter; synchronization with other physiological signals (ECG, MSNA) | Manual, event-based segmentation (computer-assisted) | Kinetic energy parameters (iKLin, iKRot) | Smirnov test and Shapiro–Wilk test (SPSS) | None reported |

Table S4. Diagnostic Performance and Reproducibility Characteristics

| Study | Evaluation Level | Sensitivity | Specificity | Accuracy / AUC / F1 | Validation Strategy | Reference standard | Data / Code Availability |
| --- | --- | --- | --- | --- | --- | --- | --- |
| [20] | Subject-level | — | — | — | Hold-out / Train-Test | PSG | No |
| [21] | Epoch-level | 93.69% | 95.30% | Accuracy 94.62% | None reported | Respiratory effort belt | No |
| [22] | Event-level | 96.70-100% | 100% | — | External validation | No | No |
| [23] | Event-level | 99.60% | 97.80% | Accuracy 98.70% | External validation | No | No |
| [24] | Epoch-level | — | — | Balanced Acc 0.527–0.920 / F1 0.508–0.916 | None reported | Nasal cannula | No |
| [25] | Event-level | — | — | — | External validation | Nasal/oral flow | No |
| [26] | Subject-level | — | — | — | None reported | Respiratory effort belt | No |
| [27] | Event-level | — | — | — | None reported | No | Yes |
| [28] | Event-level | — | — | — | External validation | No | No |
| [29] | Event-level | — | — | — | None reported | No | No |
| [30] | Epoch-level | 90% | 80% | Accuracy 85% / AUC 0.84 | 10-fold cross-validation | No | No |
| [31] | Epoch-level | — | — | — | 10-fold cross-validation | No | No |
| [32] | Subject-level | AHI>5:98% / >15:81% / >30:67% | AHI>5:36% / >15:87% / >30:94% | AUC: AHI>5:86% / >15:82% / >30:71% | 5-fold cross-validation: two loops | PSG | No |
| [33] | Event-level | — | — | — | External validation | Spirometry | No |
| [34] | Event-level | 90% | — | — | None reported | AHI Apnoelink | No |
| [35] | Subject-level | AHI>15:80% | AHI>15:100% | AccuracyAHI>15:89% | Leave-One-Out Cross Validation | PSG | No |
| [36] | Epoch-level | — | — | F1: Normal 99.3%; Hypopnoe 71.6%; OSA 82.8%; CSA 68.2% | None reported | No | No |
| [37] | Subject-level | 90% | 80% | Accuracy 85% | 10-fold cross-validation | No | No |
| [38] | Event-level | 88.60% | — | — | None reported | No | No |
| [39] | Subject-level | 40,9-80,0% | 79,4-100% | AUC: 0.71–0.91 | None reported | PSG | No |
| [40] | Epoch-level | — | 96-97% | AUROC: 87–90 % | 10-fold cross-validation | PSG | No |
| [41] | Subject-level | AHI>5:93.1% / >10:85.7% / >15:91.4% / >30:89.7% | AHI>5:56.1% / >10:83.6% / >15:89.3% / >30:98.0% | Accuracy  AHI>5:78.2% / >10:84.5% / >15:89.8% / >30:96.9% | 3-fold cross-validation | PSG | No |
| [42] | Epoch-level | — | — | — | None reported | PSG | No |
| [43] | Subject-level | — | — | — | External validation | No | No |
| [44] | Epoch-level | — | — | F1-Score: 0.75–0.914 | 10-fold cross-validation | PSG | No |
| [45] | Event-level | — | — | — | None reported | Spirometry | No |
| [46] | Event-level | — | — | — | None reported | Spirometry | No |
| [47] | Event-level | — | — | — | None reported | Spirometry | No |
| [48] | Event-level | — | — | — | None reported | Spirometry | Yes |
| [49] | Event-level | 78.14% | 100% | Accuracy 89.89% | None reported | No | No |
| [50] | Event-level | — | — | F1-Score: CSA 86 %; Breathing 95 %; Coughing 72 %; OSA 57 %; Sighing 52 %; Yawning 62 % | None reported | No | No |
| [51] | Epoch-level | — | — | — | None reported | No | No |
| [52] | Epoch-level | — | — | — | None reported | Spirometry | No |
| [53] | Event-level | — | — | — | None reported | No | No |
| [54] | Event-level | — | — | — | None reported | No | No |
| [55] | Epoch-level | — | — | — | None reported | No | Yes |
| [56] | Event-level | — | 97.50% | — | None reported | No | No |
| [57] | Event-level | — | — | — | None reported | No | No |
| [58] | Subject-level | AHI>15:91% / >30:90% | AHI>15:78% / >30:88% | Accuracy  AHI>15:85% / >30:89% | Hold-out / Train-Test | PSG | No |
| [59] | Epoch-level | 64,6-73,2% | 96,6-81,8% | Accuracy  69,5-77,9%  F1 50–57%; AUC 0,75-0,83 | Hold-out / Train-Test | PSG | Yes |
| [60] | Subject-level | — | — | — | External validation | No | Yes |
| [61] | Subject-level | — | — | — | Hold-out / Train-Test | No | No |
| [62] | Epoch-level | 30,36 % (k-NN) / 76,05 % (RUSBoost) | 75,96% (k-NN) / 70,05% (RUSBoost) | Accuracy  69,11% (k-NN) / 70,95% (RUSBoost) | 10-fold cross-validation | PSG | No |
| [63] | Subject-level | — | — | — | External validation | Spirometry | No |
| [64] | Epoch-level | — | — | — | External validation | No | No |
| [65] | Epoch-level | — | — | — | None reported | No | No |
|  |  |  |  |  |  |  |  |
