# Supplementary material for: Accelerometry-Based and Gyroscope-Based Detection of Sleep Apnea Events: Systematic Review of Technical and Clinical Evidence
Source: JMIR Biomed Eng. 2026 Oct 22;11:e104899. doi: 10.2196/104899 (PMC13717323; doi:10.2196/104899)
Supplement: Supplementary file 3 [file biomedeng-v11-e104899-s003.docx]

# Multimedia Appendix 3. QUADAS-2 Assessment.

This appendix summarizes the methodological quality assessment of all included studies using the Quality Assessment of Diagnostic Accuracy Studies 2 (QUADAS-2) tool [19]. The following domains were evaluated for each study:

1. Patient Selection
2. Index Test
3. Reference Standard
4. Flow and Timing

Each domain was assessed regarding risk of bias as “low,” “high,” or “unclear.” Applicability concerns were additionally evaluated for patient selection, index test, and reference standard domains.

Overall, the QUADAS-2 assessment revealed substantial methodological limitations across the included literature. Frequent sources of bias included:

- nonrepresentative study populations,
- simulation-based experimental designs,
- insufficient reporting of validation procedures,
- unclear or simplified reference standards,
- lack of external validation,
- incomplete reporting of patient flow and timing.

High risk of bias was particularly common in studies relying on voluntary apnea maneuvers or small cohorts of healthy participants. In contrast, studies using polysomnography-based validation in clinically representative populations generally demonstrated lower risk of bias.

Detailed study-level QUADAS-2 assessments are provided in Table S5.

Table S5. QUADAS-2 Risk of Bias Assessment.

| **Study** | **Patient Selection** | **Index Test** | **Reference Standard** | **Flow and Timing** | **Overall Risk of Bias** |
| --- | --- | --- | --- | --- | --- |
| [20] | Low | Low | Low | Low | Low |
| [21] | Unclear | Unclear | High | Low | High |
| [22] | High | High | Unclear | Unclear | High |
| [23] | Unclear | High | Unclear | Unclear | High |
| [24] | Low | Low | Low | Low | Low |
| [25] | Unclear | High | High | Unclear | High |
| [26] | High | Unclear | High | Low | High |
| [27] | Unclear | High | Unclear | Unclear | High |
| [28] | Unclear | Unclear | Unclear | High | High |
| [29] | Unclear | Unclear | Unclear | Unclear | Unclear |
| [30] | High | High | Unclear | Unclear | High |
| [31] | Unclear | High | Unclear | Unclear | High |
| [32] | Low | Low | Low | Low | Low |
| [33] | Low | Low | High | Low | High |
| [34] | High | Low | Low | Low | High |
| [35] | Low | Low | Low | Low | Low |
| [36] | Low | Unclear | Unclear | Unclear | Unclear |
| [37] | High | High | Unclear | Unclear | High |
| [38] | High | Low | Unclear | Unclear | High |
| [39] | Low | Low | Low | Low | Low |
| [40] | High | High | Low | Low | High |
| [41] | Low | Low | Low | High | High |
| [42] | Low | High | Low | Low | High |
| [43] | High | High | Unclear | High | High |
| [44] | High | Low | Low | High | High |
| [45] | High | High | High | Unclear | High |
| [46] | Low | Low | High | Low | High |
| [47] | High | High | High | Low | High |
| [48] | High | High | High | Low | High |
| [49] | Unclear | High | Unclear | Unclear | High |
| [50] | High | High | Unclear | Unclear | High |
| [51] | Unclear | High | Unclear | High | High |
| [52] | High | High | High | HIgh | High |
| [53] | Unclear | High | Unclear | Unclear | High |
| [54] | Low | High | Unclear | Unclear | High |
| [55] | Unclear | High | Unclear | Unclear | High |
| [56] | High | High | Unclear | Unclear | High |
| [57] | Unclear | Unclear | Unclear | Unclear | Unclear |
| [58] | Low | Low | Low | Low | Low |
| [59] | Low | Low | Low | Low | Low |
| [60] | High | Unclear | Unclear | High | High |
| [61] | High | Unclear | Unclear | High | High |
| [62] | High | High | Low | Low | High |
| [63] | High | High | High | High | High |
| [64] | Unclear | High | High | Unclear | High |
| [65] | High | High | Unclear | Unclear | High |
