# Supplementary material for: Accelerometry-Based and Gyroscope-Based Detection of Sleep Apnea Events: Systematic Review of Technical and Clinical Evidence
Source: JMIR Biomed Eng. 2026 Oct 22;11:e104899. doi: 10.2196/104899 (PMC13717323; doi:10.2196/104899)
Supplement: Supplementary file 4 [file biomedeng-v11-e104899-s004.docx]

# Multimedia Appendix 4. Diagnostic Performance Tables.

This appendix summarizes the reported diagnostic performance metrics of all included studies investigating accelerometry- and gyroscope-based detection of apnea and hypopnea events. Reported outcome measures included sensitivity, specificity, accuracy, area under the curve (AUC), F1-score, and apnea-hypopnea index (AHI) estimation performance where available.

Diagnostic performance was categorized according to evaluation level:

1. Event-level analysis
2. Epoch-level analysis
3. Subject-level analysis

Substantial variability in reported performance metrics was observed across studies, reflecting differences in study populations, sensor configurations, preprocessing pipelines, validation strategies, and reference standards.

Table S6. Event-Level Diagnostic Performance

| Study | Sensor Technology | Classification Task | Sensitivity | Specificity | Accuracy | AUC/F1 |
| --- | --- | --- | --- | --- | --- | --- |
| [22] | Accelerometer | Binary detection (breathing stop yes/no) | 96.7% | 100% | — | — |
| [23] | Accelerometer | Apnea detection + optional type classification | 99.60% | 97.80% | 98.70% | — |
| [25] | Accelerometer | Apnea detection via airflow cessation | — | — | — | — |
| [27] | Accelerometer | Binary event detection based on breathing intervals | — | — | — | — |
| [28] | Accelerometer | Apnea vs. non-apnea | — | — | — | — |
| [29] | Accelerometer | Binary event detection | — | — | — | — |
| [33] | Accelerometer | Apnea vs. non-apnea | — | — | — | — |
| [34] | Accelerometer | Apnea detection + AHI classes + regression | 90% | — | — | — |
| [38] | Accelerometer | Binary event detection + type classification (obstructive, central, mixed) | 88.60% | — | — | — |
| [45] | Accelerometer | Unsupervised clustering and comparison across respiratory states (inhalation, exhalation, breath-hold) | — | — | — | — |
| [46] | Accelerometer | Binary apnea detection (breathing stop yes/no) based on diaphragm movements | — | — | — | — |
| [47] | Accelerometer | Classification of different breathing patterns/movement conditions (including resting, hold, coughing, sighing, yawning) | — | — | — | — |
| [48] | Accelerometer | Not reported | — | — | — | — |
| [49] | Gyroscope and Accelerometer | Binary OSA detection | 78.14% | 100% | 89.9% | — |
| [50] | Gyroscope and Accelerometer | Six classes (normal, CSA, OSA, coughing, sighing, yawning); additionally binary models (CSA vs. normal, OSA vs. normal) | — | — | — | F1-Score: CSA 86 %; Breathing 95 %; Coughing 72 %; OSA 57 %; Sighing 52 %; Yawning 62 % |
| [53] | BCG | Binary classification (REST vs. APNEA) | — | — | — | — |
| [54] | BCG | Binary classification (REST vs. APNEA) | — | — | — | — |
| [56] | SCG | Unsupervised clustering (without clinical classes), state comparisons (normal breathing vs. breath-hold) | — | 97.50% | — | — |
| [57] | IMU | Apnea vs. non-apnea + multiclass classification (OSA vs. CSA) | — | — | — | — |

Table S7. Epoch-Level Diagnostic Performance

| Study | Sensor Technology | Epoch Duration | Sensitivity | Specificity | Accuracy | AUC/F1 |
| --- | --- | --- | --- | --- | --- | --- |
| [21] | Accelerometer | Not reported | 93.69% | 95.30% | 94.63% | — |
| [24] | Accelerometer | 12 s | — | — | Balanced Acc: 0.527–0.920 | F1: 0.508–0.916 |
| [30] | Accelerometer | 64 samples | 90% | 80% | 85% | AUC 0,84 |
| [31] | Accelerometer | Not reported | — | — | — | — |
| [36] | Accelerometer | Not reported | — | — | — | F1: Normal 99.3%; Hypopnoe 71.6%; OSA 82.8%; CSA 68.2% |
| [40] | Accelerometer | 10 s | — | 96-97% | — | AUROC: 87–90 % |
| [42] | Accelerometer | Not reported | — | — | — | — |
| [44] | Accelerometer | 20 s | — | — | — | F1: 0.75–0.914 |
| [51] | Gyroscope and Accelerometer | 10 s | — | — | — | — |
| [52] | Gyroscope and Accelerometer | 10 s | — | — | — | — |
| [55] | BCG | Not reported | — | — | — | — |
| [59] | IMU | 32 s | 64,6-73,2% | 96,6-81,8% | 69,5-77,9% | F1 50–57%; AUC 0,75-0,83 |
| [62] | IMU | 12 s | 30,36 % (k-NN) / 76,05 % (RUSBoost) | 75,96% (k-NN) / 70,05% (RUSBoost) | 69,11% (k-NN) / 70,95% (RUSBoost) | — |
| [64] | IMU | Not reported | — | — | — | — |
| [65] | BCG/SCG | Manual | — | — | — | — |
|  |  |  |  |  |  |  |

Table S8. Subject-Level Diagnostic Performance

| Study | Sensor Technology | Outcome Measure | Sensitivity | Specificity | Accuracy | AUC/F1 |
| --- | --- | --- | --- | --- | --- | --- |
| [20] | Accelerometer | AHI regression: continuous AHI estimation / apnea vs. non-apnea: binary epoch classification as an intermediate step | — | — | — | — |
| [26] | Accelerometer | Not reported | — | — | — | — |
| [32] | Accelerometer | Apnea detection + AHI severity + regression | AHI>5:98% / >15:81% | AHI>5:36% / >15:87% / >30:94% | AHI>5:78% / >15:84% / >30:88% | AUC: AHI>5:86% / >15:82% / >30:71% |
| [35] | Accelerometer | Apnea classification + AHI regression + screening | AHI>15:80% | AHI>15:100% | AHI>15:89% | — |
| [37] | Accelerometer | Binary classification (AdaBoost) using features from HRV (LF/HF) and mean values of the ACC axes | 90% | 80% | 85% | — |
| [39] | Accelerometer | Screening at AHI cutoffs | 40,9-80,0% | 79,4-100% | — | AUC: 0.71–0.91 |
| [41] | Accelerometer | Apnea detection + AHI regression + severity classification | AHI>5:93.1% / >10:85.7% / >15:91.4% / >30:89.7% | AHI>5:56.1% / >10:83.6% / >15:89.3% / >30:98.0% | AHI>5:78.2% / >10:84.5% / >15:89.8% / >30:96.9% | — |
| [43] | Accelerometer | Continuous parameter estimation (respiratory rate, etc.) | — | — | — | — |
| [58] | IMU | AHI regression + classification | AHI>15:91% / >30:90% | AHI>15:78% / >30:88% | AHI>15:85% / >30:89% | — |
| [61] | Gyroscope | Not reported | — | — | — | — |
| [63] | Accelerometer | Continuous parameter estimation (RR, —TV, phase) and regression/signal level (multivariate linear regression of the respiratory waveform) | — | — | — | — |
